# Tobacco use: prevalence and associated factors in a sample of Tunisian students

**DOI:** 10.1192/j.eurpsy.2023.1405

**Published:** 2023-07-19

**Authors:** R. Ben Soussia, F. Nour, W. Bouali, E. Gharbi, M. Ben Fredj, L. Zarrouk

**Affiliations:** 1 Psychiatry Department, Tahar Sfar Hospital Mahdia; 2 Psychiatry department, Tahar Sfar Mahdia Hospital; 3 Tahar Sfar Mahdia, Mahdia; 4 Fattouma Bourguiba Hospital, Monastir; 5 Tahar Sfar Mahdia Hospital, Mahdia, Tunisia

## Abstract

**Introduction:**

Nowadays, tobacco consumption among the student population has become a very worrying phenomenon. Given the high rate of its morbidity and mortality, knowledge of the inventory of consumption and especially tobacco addiction seems necessary.

**Objectives:**

To determine the prevalence of tobacco use and the main factors associated with it in a sample of Tunisian students.

**Methods:**

This is a descriptive and analytical cross-sectional study carried out during the 2020/2021 academic year with a sample of Tunisian students.We used an anonymous self-administered questionnaire distributed online via social networks. Our questionnaire included a section focusing on socio-demographic characteristics and the Fagerström test to detect tobacco addiction.

**Results:**

Our study enrolled 772 students .The average age of the study population was 23.29 3.25. The prevalence of tobacco consumption was 32.1%. Among the study population, 168 respondents (67.9%) were regular smokers.By evaluating our population with the Fagerström scale, 16.9% presented a strong dependence with a score >six.One hundred and six smokers (63.1%) began their smoking out of curiosity. The search for relaxation and the response to a need were the effects sought mainly (55.8% and 56.6%).The festive context and stress were the main elements increasing tobacco consumption (71% and 69.5%). The factors associated with smoking were age (p<0.005), male gender (p<10-3) and being single (p=0.035).

**Conclusions:**

Tobacco consumption represents a public health problem, particularly among young people. Measures for the prevention and management of tobacco addiction should be put in place in the university environment.

**Disclosure of Interest:**

None Declared

